# Cell Cycle Complexity: Exploring the Structure of Persistent Subsystems in 414 Models

**DOI:** 10.3390/biomedicines12102334

**Published:** 2024-10-14

**Authors:** Stephan Peter, Arun Josephraj, Bashar Ibrahim

**Affiliations:** 1 Department of Basic Sciences, Ernst-Abbe University of Applied Sciences Jena, Carl-Zeiss-Promenade 2, 07745 Jena, Germany; stephan.peter@eah-jena.de; 2 Department of Artificial Intelligence and Machine Learning, BMS Institute of Technology and Management, Bangalore 560066, India; arunjoseph.business@gmail.com; 3 Department of Mathematics & Natural Sciences and Centre for Applied Mathematics & Bioinformatics, Gulf University for Science and Technology, Hawally 32093, Kuwait; 4 Department of Mathematics and Computer Science, Friedrich Schiller University Jena, Fürstengraben, 07743 Jena, Germany; 5 European Virus Bioinformatics Center, Leutragraben 1, 07743 Jena, Germany

**Keywords:** cell cycle models, checkpoints, chemical organization theory, formal concept analysis, BioModels

## Abstract

**Background**: The regulation of cellular proliferation and genomic integrity is controlled by complex surveillance mechanisms known as cell cycle checkpoints. Disruptions in these checkpoints can lead to developmental defects and tumorigenesis. **Methods**: To better understand these mechanisms, computational modeling has been employed, resulting in a dataset of 414 mathematical models in the BioModels database. These models vary significantly in detail and simulated processes, necessitating a robust analytical approach. **Results**: In this study, we apply the chemical organization theory (COT) to these models to gain insights into their dynamic behaviors. COT, which handles both ordinary and partial differential equations (ODEs and PDEs), is utilized to analyze the compartmentalized structures of these models. COT’s framework allows for the examination of persistent subsystems within these models, even when detailed kinetic parameters are unavailable. By computing and analyzing the lattice of organizations, we can compare and rank models based on their structural features and dynamic behavior. **Conclusions**: Our application of the COT reveals that models with compartmentalized organizations exhibit distinctive structural features that facilitate the understanding of phenomena such as periodicity in the cell cycle. This approach provides valuable insights into the dynamics of cell cycle control mechanisms, refining existing models and potentially guiding future research in this area.

## 1. Introduction

Reproduction and natural selection are fundamental processes for the survival and evolution of life. During reproduction, genetic material must undergo duplication, separation, and distribution into two new daughter cells, each carrying a full complement of chromosomes and organelles. This series of events is collectively known as the cell cycle [1,2].

The cell cycle is tightly regulated and consists of distinct phases, each with specific activities and objectives [3]. These phases include the G1 phase (gap 1), which prepares the cell for DNA replication; the S phase (synthesis), during which DNA replication occurs; the G2 phase (gap 2), which prepares the cell for mitosis; and the M phase (mitotic phase), which can be further divided into several sub-phases: prophase, metaphase, anaphase, telophase, and cytokinesis [4,5,6,7]. Throughout the cycle, regulatory control mechanisms known as checkpoints ensure that the cell is ready to transition to the next phase [8,9,10,11,12].

Checkpoints are controlled by various proteins, including cyclins and cyclin-dependent kinases (CDKs), which prevent the cycle from proceeding if certain conditions are not met, such as incomplete DNA replication or DNA damage [1]. The cell cycle plays a crucial role in various biological processes, and its dysfunction can lead to diseases such as cancer [13,14]. Understanding the cell cycle is therefore of both fundamental and medical importance [14,15,16,17].

In computational biology, various mathematical frameworks are employed to model the cell cycle to understand its dynamics, regulation, and checkpoints [9,18,19,20,21,22]. These models are often validated using experimental data, which facilitates hypothesis testing and the prediction of mutation or drug effects and deepens our understanding of the fundamental principles governing the cell cycle [9,23,24,25]. Models aimed at capturing the essence of the biological processes involved in the cell cycle often involve complex equations or simulations [9,26,27,28,29,30,31,32,33,34,35].

Conventional methodologies in computational biology for exploring the cell cycle include ordinary differential equations (ODEs) [36,37,38,39,40,41,42,43,44], partial differential equations (PDEs) [27,45,46,47,48,49,50], and stochastic models [29,51,52,53,54], among others. However, these methods often require kinetic data, such as reaction rates or diffusion constants, which can be challenging to obtain experimentally and computationally. Moreover, solving high-dimensional systems numerically can be infeasible [49,55,56,57,58,59].

An alternative approach, known as chemical organization theory (COT), offers a different perspective. Designed primarily for constructive systems, COT relates the structure of a reaction network to the potential dynamics of the associated dynamical system [60,61,62]. This method enables the analysis and understanding of a broad range of dynamical systems based on reaction networks. By employing the COT, the internal structure of these models can be uncovered, allowing them to be classified and compared within a common framework.

Recent advancements have expanded COT’s applicability. Initially, it was established that each fixed point within an ordinary differential equation (ODE) system corresponded to an organization as defined by the COT [60,63]. Recent developments have included incorporating spatial and temporal distributions into the COT, exemplified by the concept of distributed organizations (DOs) [64]. Additionally, persistence in reaction–diffusion systems has been addressed [65,66,67]. These advancements enhance COT’s mathematical framework and extend its application to complex dynamical systems.

In this study, we analyze 414 models using the COT to explore their compartmentalized structures and dynamic behaviors. Our goal is to identify distinctive features that reveal new insights into cell cycle mechanisms and to provide a framework that enhances the understanding of these complex systems. This approach not only refines existing models but also guides future research by highlighting key areas for further investigation.

## 2. Materials and Methods

The methodology begins with establishing the groundwork through Preliminaries, where fundamental concepts and definitions are introduced. Next, we explore the concept of “Organizations” from the perspective of the chemical organization theory (COT). We then explain how these organizational structures relate to the behavior of dynamic systems. Finally, we conduct a meticulous examination of the parameters governing the cell cycle models.

To enhance accessibility to the mathematical aspects, we consistently use a straightforward example throughout the Methods section. This example is drawn from a well-known 6-variable cell cycle model introduced by Tyson [37] in 1991. It provides a clear and illustrative framework for explaining various mathematical principles and methodologies discussed in this section. Tyson’s model, one of the earliest to illustrate cell physiology through molecular concentrations, employs differential equations to describe the cell-division cycle based on the concentrations of proteins cdc2 and cyclin. The model accounts for the formation of protein complexes and their subsequent phosphorylation and dephosphorylation.

By theoretically adjusting parameter values that govern the activation rate of the cyclin–Cdc2 complex, known as maturation-promoting factor (MPF), and its dissociation rate, the model exhibits three distinct behaviors: a stable state with high MPF levels (analogous to metaphase arrest in unfertilized eggs), spontaneous oscillations (representing rapid division cycles in embryonic cells), and an excitable switch (depicting growth-controlled division in proliferating cells).

Finally, the organizational structure of a second cell cycle model, consisting of 16 variables and 27 reactions as introduced by Markevich in 2004 [68], is analyzed. This discussion should provide a comprehensive explanation of the methodology employed in this work, supported by two detailed examples for clarity.

### 2.1. Preliminaries

A **reaction network** is a set of species together with a set of reactions according to which the species interact together. All reaction networks studied in this work were taken as SBML models from the BioModels Database [69]. The reaction network of Tyson’s paper used m=9 species and n=9 reactions. The species were

$$\begin{array}{cc} C2: & \;\mathrm{cdc}2\;\left(\mathrm{enzyme}\right)\\ CP: & \;\mathrm{cdc}2\text{-}P\;(\mathrm{phosphorylated}\;\mathrm{cdc}2)\\ pM: & \;\mathrm{preMPF}\;=\;P\text{-}\mathrm{cyclin}\text{-}\mathrm{cdc}2\text{-}P\;\mathrm{complex}\;(\mathrm{MPF}\;=\;“\mathrm{maturation}\;\mathrm{promoting}\;\mathrm{factor}”)\\ M: & \;\mathrm{active}\;\mathrm{MPF}\;=\;P\text{-}\mathrm{cyclin}\text{-}\mathrm{cdc}2\\ Y: & \;\mathrm{cyclin}\;\left(\mathrm{protein}\right)\\ YP: & \;\mathrm{cyclin}\text{-}P\;(\mathrm{phosphorylated}\;\mathrm{cyclin})\\ aa: & \;\mathrm{amino}\;\mathrm{acids}\;\left(\mathrm{constant}\right)\\ \;P: & \;\mathrm{ATP}\;\left(\mathrm{constant}\right)\\ P_{i}: & \;\mathrm{inorganic}\;\mathrm{phosphate}\;\left(\mathrm{constant}\right)\\ Ø: & \mathrm{EmptySet},\;\mathrm{used}\;\mathrm{as}\;a\;\mathrm{species}\;\mathrm{only}\;\mathrm{in}\;\mathrm{the}\;\mathrm{SBML}\;\mathrm{model}\;\mathrm{instead}\;\mathrm{of}\;aa,\;\sim P,\;P_{i} \end{array}$$

The constant species $aa,\;\sim P,\;P_{i}$ used in Tyson’s paper to model in- and outflows were replaced by the EmptySet in the SBML model. The reactions of Tyson’s model were

$$\begin{array}{cc} R_{1}: & \;Ø\;\overset{k_{1}}{\to }\;Y\;(\mathrm{SBML}\;\mathrm{Reaction}6:\;\mathrm{de}\;\mathrm{novo}\;\mathrm{synthesis}\;\mathrm{of}\;\mathrm{cyclin})\\ R_{2}: & \;Y\;\overset{k_{2}}{\to }\;Ø\;(\mathrm{SBML}\;\mathrm{Reaction}7:\;\mathrm{cyclin}\;\mathrm{might}\;\mathrm{be}\;\mathrm{unstable})\\ R_{3}: & \;CP+Y\overset{k_{3}}{\to }pM\;(\mathrm{SBML}\;\mathrm{Reaction}4:\;\mathrm{cdc}2\text{-}P\;\mathrm{combines}\;\mathrm{with}\;\mathrm{cyclin}\;\mathrm{to}\;\mathrm{preMPF}\;\&\;\mathrm{phosphorylation})\\ R_{4}: & \;pM\overset{k_{4}}{\to }M\;(\mathrm{SBML}\;\mathrm{Reaction}9:\;\mathrm{dephosphorylation}\;\mathrm{of}\;\mathrm{cdc}2\;\mathrm{to}\;\mathrm{form}\;\mathrm{active}\;\mathrm{MPF})\\ R_{5}: & \;M\overset{k_{5}}{\to }pM\;(\mathrm{SBML}\;\mathrm{Reaction}5:\;\mathrm{opposition}\;\mathrm{of}\;\mathrm{MPF}\;\mathrm{activation}\;\mathrm{by}\;a\;\mathrm{protein}\;\mathrm{kinase})\\ R_{6}: & \;M\overset{k_{6}}{\to }C2+YP\;(\mathrm{SBML}\;\mathrm{Reaction}1:\;\mathrm{destruction}\;\mathrm{of}\;\mathrm{active}\;\mathrm{MPF}\;\mathrm{releasing}\;\mathrm{phosphorylated}\;\mathrm{cyclin})\\ R_{7}: & \;YP\overset{k_{7}}{\to }Ø\;(\mathrm{SBML}\;\mathrm{Reaction}8:\;\mathrm{rapid}\;\mathrm{proteolysis}\;\mathrm{of}\;\mathrm{phosphorylated}\;\mathrm{cyclin})\\ R_{8}: & \;C2\overset{k_{8}}{\to }CP\;(\mathrm{SBML}\;\mathrm{Reaction}2:\;\mathrm{phosphorylation}\;\mathrm{of}\;\mathrm{cdc}2)\\ R_{9}: & \;CP\overset{k_{9}}{\to }C2\;(\mathrm{SBML}\;\mathrm{Reaction}3:\;\mathrm{possibly}\;\mathrm{reversion}\;\mathrm{of}\;R_{8}) \end{array}$$

**Figure 1:**
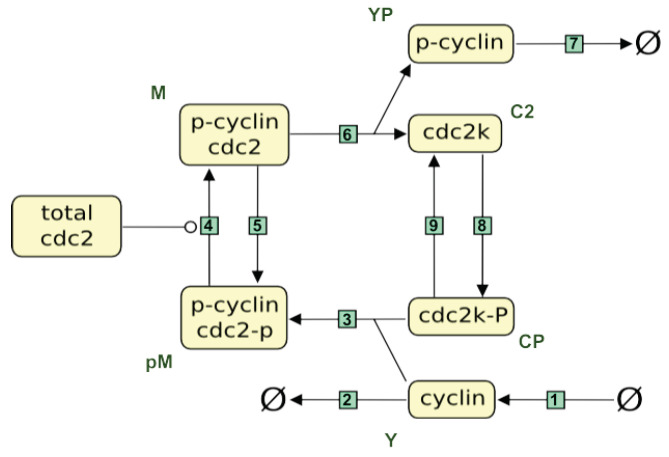
A biochemical reaction network illustrating the interactions and transitions between cyclin, cdc2, and their phosphorylated states in cell cycle regulation according to the paper [37]. The plot was obtained from the EBI Biomodels website. The proteins cdc2 (C2, phosphorylated: C2P) and cyclin (Y, phosphorylated: YP) form a heterodimer (maturation-promoting factor) P-cyclin–cdc2 (and P-cyclin–cdc2-P) that controls the major events of the cell cycle. The numbers inside the square green box denote the reaction numbers.

The reaction numbering on the left refers to Tyson’s paper, whereas the numbers of the reactions used in the SBML model are given in brackets after the reaction equations. The species on the left-hand side of the arrow of a reaction equation are called **reactants** whereas those on the right-hand side are called **products**. Figure 1 shows Tyson’s diagram [37], which visualizes the reaction network.

From the reaction equations, the **stoichiometric matrix** $N\in R^{m\times n}$ can be computed. For Tyson’s model, with the numbering from the SBML model, it is

(1) $$\begin{array}{c} N=\left(\begin{array}{ccccccccc} 1 & -1 & 1 & 0 & 0 & 0 & 0 & 0 & 0\\ 0 & 1 & -1 & -1 & 0 & 0 & 0 & 0 & 0\\ 0 & 0 & 0 & 1 & 1 & 0 & 0 & 0 & -1\\ -1 & 0 & 0 & 0 & -1 & 0 & 0 & 0 & 1\\ 0 & 0 & 0 & -1 & 0 & 1 & -1 & 0 & 0\\ 1 & 0 & 0 & 0 & 0 & 0 & 0 & -1 & 0 \end{array}\right)\in Z^{6\times 9} \end{array}$$

Each column of *N* corresponds to a reaction, and each line to one of the first six species given in the list of species above (the empty set is neglected because it is constant). If an entry of *N* is zero, the corresponding species is not affected by the respective reaction. On the other hand, if the species is affected, then the non-zero entry is the difference in appearances of that species among the products of the reaction minus the number of appearances among the reactants. For example, Reaction1 consumes 0−1=−1*M* and produces 1−0=1 of each C2 and YP, which leads to the three non-zero entries in the first column of *N*.

For a given subset of species, a **set of active reactions** contains those for which the **support**, that is, the set of its reactants, is a subset of it. An exception of this are inflow reactions like Reaction6 (the set of reactants is empty), which can but do not necessarily belong to the set of active reactions that is uniquely defined for any subset of species. For example, for the subset {CP,Y}, the sets are {Reaction6,Reaction3,Reaction4,Reaction7} and {Reaction3,Reaction4,Reaction7}. For a given subset *S* of species, a vector v$\in R_{+}^{n}\equiv {\left\{\alpha \in R:\;\alpha \geq 0\right\}}^{n}$ is called **feasible flux** if there is a set *R* of active reactions such that

(2) $$\begin{array}{c} v_{j}>0\Leftrightarrow \;R_{j}\in R. \end{array}$$

For example, for the subset {CP,Y}, the vectors $v^{T}=\left(0,0,1,1,0,1,1,0,0\right)$ and $w^{T}=\left(0,0,1,1,0,0,1,0,0\right)$ are feasible fluxes, one for which the inflow Reaction6 is active, and one for which it is not.

### 2.2. Organizations in the Sense of Chemical Organization Theory (COT)

Chemical organization theory is a branch of reaction network analysis studying subnetworks, so-called **organizations** [70]. An **organization** is a subnetwork of the reaction network comprising a subset *S* of species and a subset *R* of active reactions with respect to *S*. It fulfills the following two properties:

1. **Closedness**, that is, no species or reaction is created or activated, which is not already contained in the organization;
2. **Self-preservation**, that is, the subsystem can operate in an equilibrium state, in which it can compensate for all species that are consumed. That is, there is a feasible flux $v\in R_{+}^{n}$ with respect to *S*, such that for all species s∈S, the following holds:
  (3) $$\begin{array}{c} {\left(N\cdot v\right)}_{s}\geq 0. \end{array}$$

This is the case of an organization consisting of only one compartment, where all species are mixed together. In general an organization can be **distributed** or **compartmentalized** [64,66], that is, it can have any number of k∈N pairwise disjoint compartments $S_{i}⫅S$ with their subsets $R_{i}$ of active reactions such that

- $S=\cup_{i=1}^{k}S_{i}$ and $R=\cup_{i=1}^{k}R_{i}$;
- Each $S_{i}$ is closed;
- There is a feasible flux $v\in R_{+}^{n}$ with ${\left(N\cdot v\right)}_{s}\geq 0$ for all s∈S.

As shown below, COT allows for deriving statements about the behavior of dynamical systems from the structure of its underlying reaction network. Therefore, in this study, organizations are oriented on their active reactions rather than their species, which include so-called non-reactive species that are not interesting with regard to the dynamics. Nevertheless, for the set of active reactions of any organization, a set of (reactive) species is uniquely determined by assuming **minimal compartmentalization**, that is, one with as few as possible compartments.

**Figure 2:**
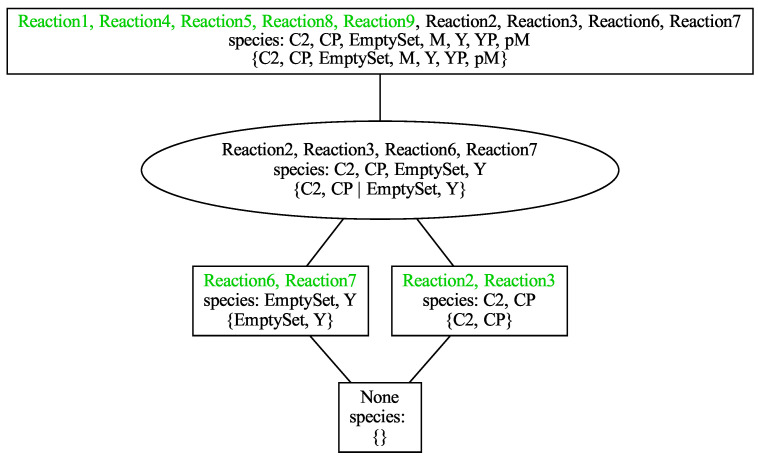
Lattice of organizations of the reaction network of Tyson’s model [37] with reaction numbering according to the SBML model in the BioModels database [69]. An illustrative representation of the organizations as subnetworks of the reaction network follows in Figure 4.

For Tyson’s model, all organizations are depicted in Figure 2, showcasing their sets of active reactions, corresponding species, and a minimal compartmentalization. They are arranged in a lattice, a configuration possible for every reaction network [64].

Each node of the lattice represents an organization. The text within each node contains three lines, which, from top to bottom, display the reactions, the species, and a **minimal compartmentalization**, that is, one with the least number of compartments. In the vertical direction, the organizations are arranged increasingly in the lattice from bottom to top according to their number of reactions. Two organizations of different vertical levels are linked by a line if and only if the set of reactions of the lower one is a subset of the set of reactions of the upper one. If a reaction of an organization appears for the first time, that is, it does not appear in any of its subsets, then it is displayed in green color. As mentioned above, the third line of each node displays its compartmentalization. More precisely, the compartments are separated by a vertical line |. An organization is bounded by a rectangular frame, if it has a compartmentalization consisting of only one compartment. Otherwise, it is bounded by an ellipse and requires compartmentalization to be an organization. To list and describe the five organizations of Tyson’s model, species name abbreviations are used instead of the reaction numbers, as they are more descriptive:

- $O_{0}=\left\{\right\}$: The empty set is the smallest organization for all models since inflow reactions are considered optional with respect to the empty species set, as mentioned above.
- $O_{1}=\left\{\mathrm{EmptySet},\;Y\right\}$: the only two active reactions are the inflow Reaction6 producing *Y* and its inverse outflow Reaction7.
- $O_{2}=\left\{C2,\mathrm{CP}\right\}$: it involves two organizations within a single compartment, allowing for the activation of the two active inverse reactions, Reaction2 and Reaction3.
- $O_{3}=\left\{C2,\mathrm{CP}\;|\;\mathrm{EmptySet},Y\right\}$: It represents a kind of trivial disjoint union of its two sub-organizations, which is possible for any number of organizations because no new reactions are activated in this manner. Since in the lattice, for every organization, a minimal compartmentalization is given and there is no organization with the same species merged in a single compartment, it is clear that at least one new reaction would be activated by doing so, which would either destroy the closedness or the self-preservation or both. A look at the reactions reveals that Reaction4 destroys the closedness by producing the new species pM and in turn a cascade of further species leading to further reactions. This stops only after all species and reactions of the model are involved, which constitute an organization once again: $O_{4}$.
- $O_{4}=\left\{C2,\mathrm{CP},\mathrm{EmptySet},\mathrm{YP},\mathrm{pM}\right\}$: The two disjoint organizations $O_{1}$ and $O_{2}$ can constitute an organization when merged but not without the two newly produced species pM and YP and the five newly activated reactions. Thus, the total reaction network can persist, or conversely, no species or reaction is predetermined through the reaction network to vanish. In other words, the set difference of the total reaction network minus the biggest organization is the empty set with regard to species as well as reactions.

### 2.3. The Relation between Organizations and the Behavior of Dynamical Systems

In this work, a dynamical system is defined as a set of differential equations of the form:

(4) $$\begin{array}{c} \frac{\partial }{\partial t}c_{i}\left(x,t\right)=\underset{\mathrm{reactions}\;}{\underset{⎵}{Nv(c\left(x,t\right))}}+\underset{\mathrm{diffusion}\;}{\underset{⎵}{d_{i}\frac{\partial^{2}}{\partial x^{2}}c_{i}\left(x,t\right),}}\;i=1,\ldots ,n,t\geq 0, \end{array}$$

where $c_{i}\left(x,t\right)$ is the concentration of the species $s_{i}$ at the location *x* at time *t*, $\frac{\partial }{\partial t}$ and $\frac{\partial^{2}}{\partial x^{2}}$ are the (partial) first and second derivatives of $c_{i}\left(x,t\right)$, resp., with respect to *x* and *t*, resp., *N* is the stoichiometric matrix of the underlying reaction network, v() is a flux vector function producing feasible vectors with respect to any c(x,t), and $d_{i}\in R_{+}$ are the so-called diffusion rates. If all diffusion rates equal zero and $c_{i}$ does not depend on any spatial variable, then the diffusion term vanishes, the partial derivatives become usual derivatives, and this PDE system, also called **reaction–diffusion system**, shrinks to an **ODE system**. This is the case for the ODE system described in Tyson’s paper [37] (see below).

By exploring the relationship between the dynamics of partial differential equation (PDE) or ordinary differential equation (ODE) systems and the structure of the underlying reaction network, a novel refined concept of **persistence** naturally emerges. Persistence is defined as a mathematically precise identification of the subsets of species or reactions that consistently maintain strictly positive, non-vanishing concentrations or fluxes throughout all time intervals [64,66]. This concept allows us to find the answer, which represents one of the most important theorems of the COT [64,66]:

The set of persistent species, resp., reactions of a dynamical system (4) always represents an organization of the underlying reaction network.

It follows that every subset of species or reactions which is not equal to an organization must vanish at some time in every solution of the dynamical system. Even though all organizations can be computed solely from the reaction equations underlying a dynamical system, they encapsulate every potentially persistent subnetwork of any solution. This includes all scenarios, such as various reaction rates, initial conditions, diffusion rates, and diverse dynamics like fixed points, periodic orbits, heteroclinic orbits, chaotic behavior, among others. And this holds true only when the species of an organization are allowed to distribute into different compartments, which might be separated from one another in time, in space, or functionally, for example, through the action of a membrane.

The solutions of the ODEs in Tyson’s model can be expressed as follows:

$$\begin{array}{cc} \frac{d[C2]}{dt} & =k_{6}\left[M\right]-k_{8}\left[\sim P\right]\left[C2\right]+k_{9}\left[\mathrm{CP}\right]\\ \frac{d[\mathrm{CP}]}{dt} & =-k_{3}\left[\mathrm{CP}\right]\left[Y\right]+k_{8}\left[\sim P\right]\left[C2\right]-k_{9}\left[\mathrm{CP}\right]\\ \frac{d[\mathrm{pM}]}{dt} & =k_{3}\left[\mathrm{CP}\right]\left[Y\right]-\left[\mathrm{pM}\right]F\left(\left[M\right]\right)+k_{5}\left[\sim P\right]\left[M\right]\\ \frac{d[M]}{dt} & =\left[\mathrm{pM}\right]F\left(\left[M\right]\right)-k_{5}\left[\sim P\right]\left[M\right]-k_{6}\left[M\right]\\ \frac{d[Y]}{dt} & =k_{1}\left[\mathrm{aa}\right]-k_{2}\left[Y\right]-k_{3}\left[\mathrm{CP}\right]\left[Y\right]\\ \frac{d[\mathrm{YP}]}{dt} & =k_{6}\left[M\right]-k_{7}\left[\mathrm{YP}\right] \end{array}$$

which function in one of three modes:

1. As a steady state with high maturation promoting factor activity (associated with metaphase arrest in unfertilized eggs);
2. As a spontaneous oscillator (rapid division cycles in early embryos);
3. As a excitable switch (growth-controlled division cycles typical of nonembryonic cells).

**Figure 3:**
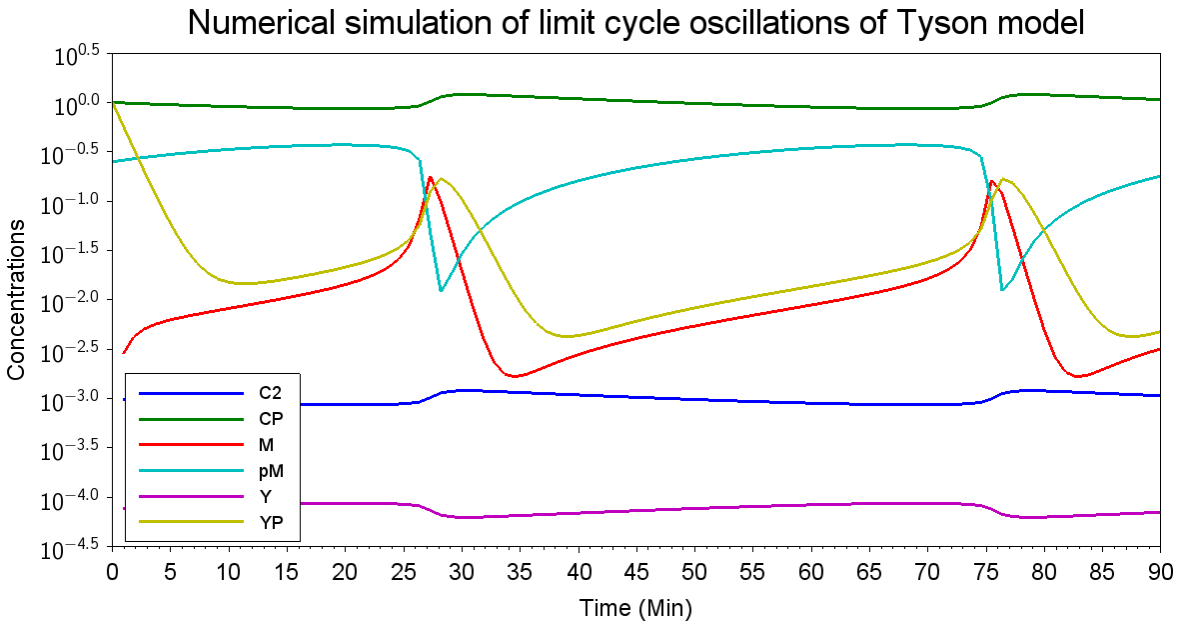
The numerical simulation of limit cycle oscillations in Tyson’s 1991 model [37] illustrates the log-scaled dynamic concentrations of Cdc2 (C2), the cyclin–Cdc2 complex (CP), phosphorylated cyclin–Cdc2 (pM), cyclin (Y), and phosphorylated cyclin (YP) over a period of 90 min. This visualization highlights the regulatory feedback mechanisms that drive cell cycle progression. The initialization values for the variables C2, CP, M, PM, Y, and Yp were 0, 0, 1, 0, 0.25, 0, and 1, respectively. The reaction constants were k_1_ = 0.015, k_2_ = 0, k_3_ = 200, k_4_ = 180, k_5_ = 0, k_6_ = 1, k_7_ = 0.6, k_8_ = 1,000,000, and k_9_ = 1000.

An example simulation result for Tyson’s model exhibiting limit cycle oscillations is depicted in Figure 3. This simulation was conducted using Scilab (2024.1.0).

### 2.4. Parameters of the Cell Cycle Models

The methods introduced above enabled the derivation of a parameter list for each cell cycle model analyzed in this study. This facilitated a concise description of each model and enabled the comparison in the Results section. This technique was previously demonstrated [66] and partially applied to models of viral infection dynamics, including influenza [64], and SARS-CoV-2 [65]. The parameters, along with their values for Tyson’s model and a description, were as follows:

- **Reaction network complexity**
  This referred to the number of species and the number of reactions in the models
- **Organizational complexity**
  The parameters included the number of organizations and reactions of a model, as well as the height and width of the lattices. Additionally, the persistence of the total network was considered, which was defined as the number of reactions in the largest organization (located at the top of the lattice) divided by the total number of reactions *n*; the maximum value was 1, indicating that the entire network was persistent.
- **Complexity of Compartmentalization**
  This metric assessed the fraction of organizations that necessitated more than one compartment and the maximum number of compartments required by an organization.
- **Time complexity**
  The total runtime to compute the lattice of organizations (in milliseconds) is provided. For example, in Tyson’s six-variable model, the value was 0.0265ms.

In the next two sections, we determine and interpret these parameters for both Tyson’s model [37] and Markevich’s model [68]. Tyson’s model, which is relatively small with 9 species and 9 reactions, is contrasted with the larger and more complex Markevich model, which includes 16 variables and 27 reactions. Each model is analyzed in detail.

### 2.5. Exemplary Organizational Analysis of Tyson’s Model

**Figure 4:**
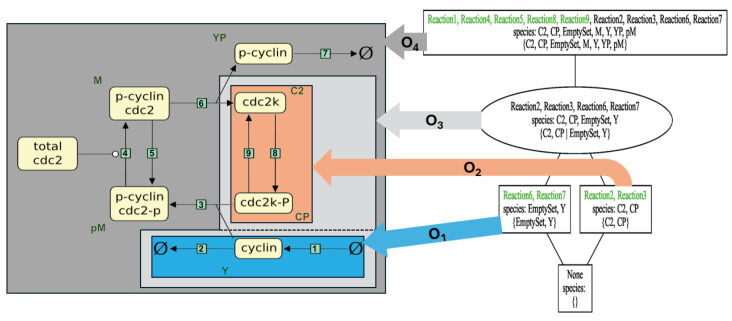
(**Left**) Reaction network of Tyson’s model [37] overlaid with a Venn diagram depicting the various (non-empty) organizations. (**Right**) Lattice of organizations from Figure 2, with arrows indicating the corresponding subsystems within the reaction network shown on the left.

Figure 4 displays the organizations of Tyson’s model as subsystems within the reaction network graph. This visual representation highlights the interactions and dependencies among the 9 species and 9 reactions, offering insight into the model’s structural and functional dynamics. In Tyson’s model of the cell cycle, the entire reaction network corresponds to the organization $O_{4}$. This indicates that the entire reaction network can persist dynamically. This is demonstrated by the simulation results shown in Figure 3, which exhibit persistent periodic behavior with all species maintaining strictly positive concentrations.

Furthermore, Figure 4 shows three proper subsystems of $O_{4}$, which can each persist on their own too. These are the two organizations $O_{1}$ (synthesis and decay of cyclin) and $O_{2}$ (phosphorylation of cdc2 and reversion), which can each persist alone or coexist side-by-side (represented by $O_{3}$) as long as they are not mixed. If they are mixed, Reaction3 initiates a chain of reactions producing the whole system $O_{4}$. There, the dynamics can settle or fall down to one of its four proper subsystems $O_{0}$ to $O_{3}$.

Figure 3 reveals the synchronization between $O_{1}$ (cyclin) and $O_{2}$ (Cdc2 and Cdc2-P) due to their mutual influence. Additionally, within $O_{2}$, a positive correlation is observed between the concentrations of Cdc2 (blue line) and Cdc2-P (green line). Conversely, an anti-correlation is noted between these species and $O_{1}$.

Altogether, this exemplifies that a persistent (sub)system of the cell cycle is always an organization. Furthermore, it demonstrates that each “sub-organization” exhibits internal coherence, which may not necessarily be present between different sub-organizations.

Now, the parameters introduced in Section 2.4 can be easily derived and understood for Tyson’s model (see Figure 2). For the organizational complexity and the complexity related to compartmentalization, they are as follows:

- A height of four, that is, with regard to size, there are four different levels of persistent subsystems of this cell cycle model;
- A width of two, that is, there are at most two different persistent subsystems on one and the same level, which can coexist;
- A persistence of 1, that is, the system as a whole is persistent;
- A fraction of organization requiring compartmentalization of 1/5;
- A maximum number of required compartments of two, that is, if the model allows for at least two distinct compartments, it exhibits one more persistent subsystem than without compartmentalization.

### 2.6. Exemplary Organizational Analysis of Markevich’s Model

**Figure 5:**
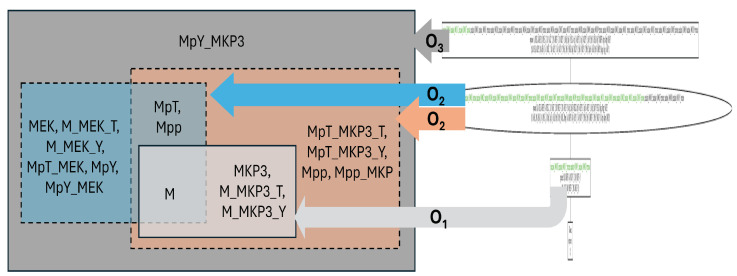
Venn diagram (**left**) and lattice (**right**) of organizations in Markevich’s model [68]. There are four non-empty organizations. From the smallest to the biggest, these are $O_{1}$ (light gray) containing four species; $O_{2}$, which contains at least two compartments, one that is colored blue (left) and one that is colored orange (right) and includes all of $O_{1}$ and a blue-colored one; and finally $O_{3}$ (dark gray), which is the biggest one and includes the whole system.

Figure 5 displays the Venn diagram and lattice of organizations for Markevich’s model [68], which focuses on mitogen-activated protein kinase (MAPK) cascades. The lattice is extensive, highlighting its structural complexity.

MAPK cascades are regulatory elements that modulate the cell cycle’s progression by responding to various stimuli, controlling checkpoint pathways, and regulating gene expression necessary for cell division. The four organizations of Markevich’s model form a chain of increasing subsystems. The smallest organization, $O_{0}=\left\{\right\}$, is trivial, as noted in the description of Tyson’s model. As in Tyson’s model, the largest organization, $O_{3}$, is the entire reaction network, where all components can coexist without any compartmentalization. The same holds true for the smallest non-empty organization, $O_{1}$, which contains only four species. $O_{2}$ lies between $O_{1}$ and $O_{3}$. Its set of species and reactions differs from those in $O_{1}$ and $O_{3}$ and can only exist when the system is separated into two compartments. Otherwise, $O_{2}$ would not be closed, as the binding of MpY and MKP3 would produce MpY_MKP3, which is not included in $O_{2}$.

Now the parameters for the organizational complexity and the complexity related to compartmentalization can be determined:

- A height of four, that is, with regard to size, there are four different levels of persistent subsystems of this cell cycle model (as for Tyson’s model);
- A width of one, meaning that there is only one persistent subsystem at each level.
- A persistence of 1, indicating that the system as a whole is persistent, similar to Tyson’s model.
- A fraction of organization requiring compartmentalization of 1/4;
- A maximum number of required compartments of two, meaning that an organization can persist only if its species are separated into two distinct compartments

In summary, Markevich’s model, unlike Tyson’s model, does not feature two distinct persistent subsystems that are not subsets of each other. However, similar to Tyson’s model, Markevich’s model contains a subsystem that can only maintain its persistence by separating its species into at least two subsets that are not subsets of each other. Nonetheless, these two subsets themselves are not persistent in Markevich’s model.

## 3. Results

**Figure 6:**
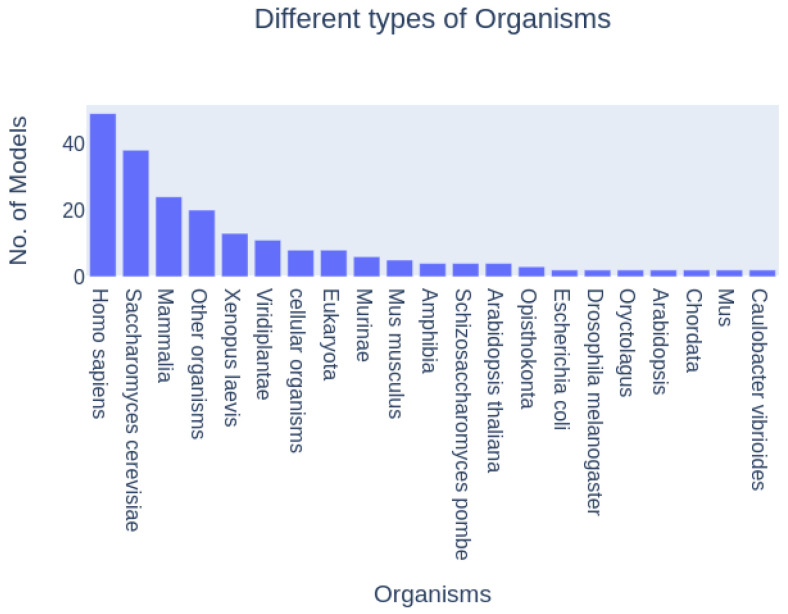
Histogram of cell cycle models categorized by the organisms studied. Some models overlap, particularly those addressing transitions such as S/G2 or G2/M. The majority of the models focus on the M phase, with approximately 160 dedicated to that stage.

This work is a meta-study that aims to demonstrate, through the organization-theoretical investigation of cell cycle models of different organisms, stages, and parts of the cell cycle, the universally applicable possibilities offered by reaction network analysis and in particular chemical organization theory (COT) to support modeling of the cell cycle. Figure 6 gives an overview of how the models studied are distributed across the organisms involved.

### 3.1. Distribution of Parameters across Cell Cycle Models

**Figure 7:**
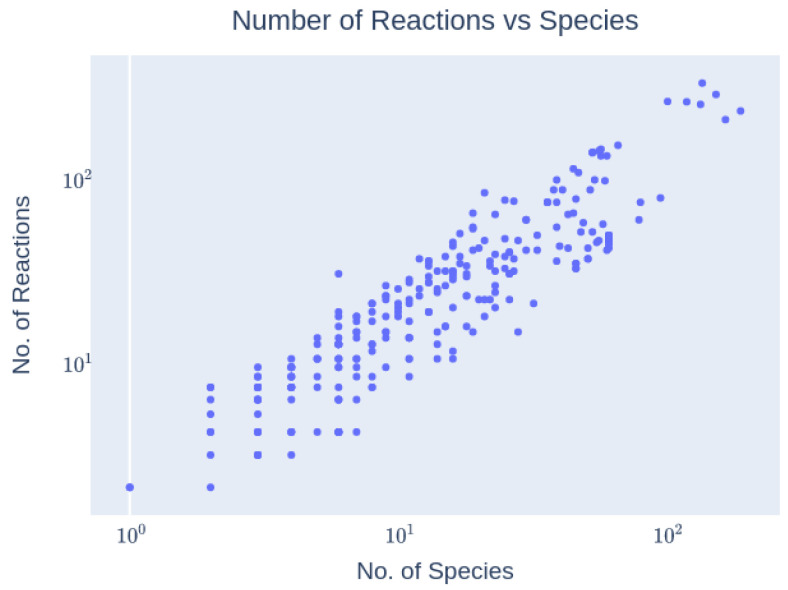
Reaction network complexity: Scatter plot of the number of species vs. the number of reactions of each model. As expected, there is an overall positive correlation between the two. The number of species ranges from 1 to 189, and the number of reactions from 2 to 316.

In this section, we analyze the distribution of key parameters across all 414 cell cycle models. The distribution is graphically illustrated in Figure 7, which depicts the range and frequency of parameter values across different model categories. The analysis reveals significant variability in the parameters, with notable concentrations in certain ranges. This variability highlights the diverse nature of the models included in our study and provides a foundation for understanding the impact of these parameters on model behavior.

The analysis examined a total of 414 models from the BioModels database, showcasing a wide spectrum of diversity. These models varied significantly in size and complexity, ranging from those featuring a few species to several hundred, encompassing various reactions and processes (see Figure 7).

This comprehensive overview underscores the central role of well-established model organisms in cell cycle studies while also pointing to opportunities for expanding research to underrepresented species.

### 3.2. Organizational Complexity

**Figure 8:**
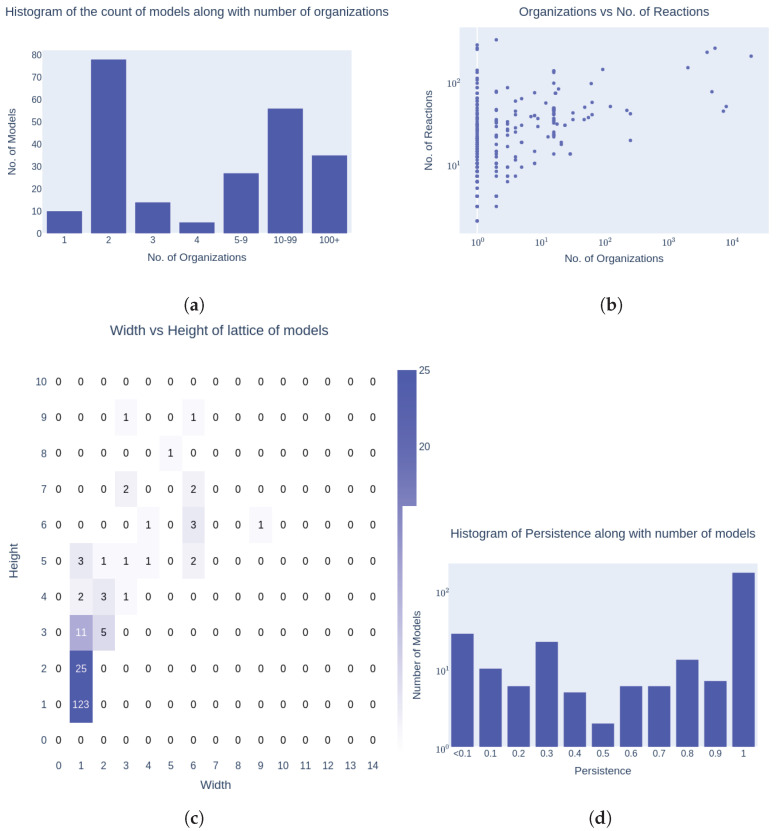
Lattice of organizations’ complexity: (**a**) Histogram of the number of models according to their number of organizations and (**b**) scatter plot of the number of organizations vs. the number of reactions. The most frequent number of organizations per model was two. The model size with regard to the number of reactions was not strongly connected to the number of organizations. (**c**) Height vs. width scatter plot, with values of each data point given by color. To better represent the correlation, two models were removed by cutting the diagram above a width of 10: one with a width of 56 and a height of 8, and another with a width of 20 and a height of 7. Additionally, 39 models with no species and 10 very large models in their Hasse diagram were excluded from the plot. In total, the full lattice of organizations was calculated for 275 models within the pre-limited time. (**d**) Histogram showing the values of persistence of different models. (**a**) Histogram of the model frequencies according to their number of organizations; (**b**) Number of organizations vs. number of reactions; (**c**) Height vs. width scatter plot, with values of each data point represented by color; (**d**) Histogram showing the values of persistence of different models.

The organizational structure within these models exhibited notable diversity. Some models were characterized by a simple arrangement, with just one or two organizations, while others featured intricate networks with over a hundred organizations (see Figure 8).

Measured by the number of organizations per model, the models with two or more organizations appeared less frequently the more organizations they had, see the histogram in Figure 8a, noting that the class width in the histogram increased starting from five organizations per model. This observation underscores the dynamic nature of organizational configurations within cellular models. Additionally, Figure 8b demonstrates that while a certain number of reactions was necessary to achieve a specific level of organizational structure within a model, a high number of reactions did not always guarantee a high number of organizations. This indicated a complex relationship between reaction count and organizational complexity in cellular models.

Figure 8c reveals that a specific width of a lattice could only be attained with a certain height, but a certain height did not depend on the width. In relation to the cell cycle, this means that a consistent parallel operation of various subsystems is only possible if there are a sufficient number of finely subdivided levels of subsystems. This finding sheds further light on the intricate interplay between structural dimensions within these models.

Most models were structured to maintain overall persistence, although there were instances where persistence varied (see Figure 8d). There were examples of varying degrees of persistence, where the largest persistent subsystem represented only a fraction of the entire system. This highlights the diversity in model dynamics and the range of persistence levels observed within cellular models. It also suggests that the modeling primarily aims at simulating dynamic behavior, while neglecting the development of a consistent, widely accepted basic structure into which all models can be embedded.

### 3.3. Compartmentalization Complexity

**Figure 9:**
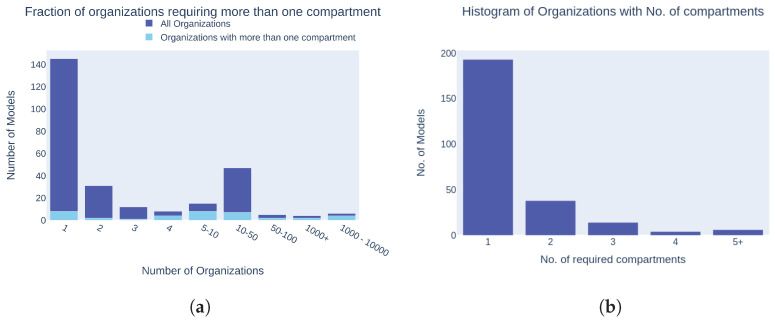
Compartmentalization complexity: (**a**) Comparison of organizations with only one compartment versus those requiring more than one compartment. (**b**) Histogram showing the count of the maximum number of required compartments in an organization. (**a**) Number of models with organizations all containing only one compartment vs. models containing at least one organization requiring more than one compartment; (**b**) histogram of required compartments across models.

The analysis of compartmentalization complexity in cell cycle models revealed that the majority of models (nearly 140) involved organizations with only one compartment, as shown in Figure 9a.

Models requiring multiple compartments were less common, with a significant drop in frequency as the number of compartments increased. Figure 9b supports this observation, indicating that most models required just one compartment, while a smaller number necessitated two, and very few required three or more compartments. This trend suggests that simpler, single-compartment models are predominantly used in cell cycle research, possibly due to their lower complexity and ease of analysis. However, the presence of multi-compartment models highlights the necessity to capture more intricate cellular processes in certain studies, pointing to an area where increased complexity might be essential for accurate representation.

### 3.4. Time Complexity

**Figure 10:**
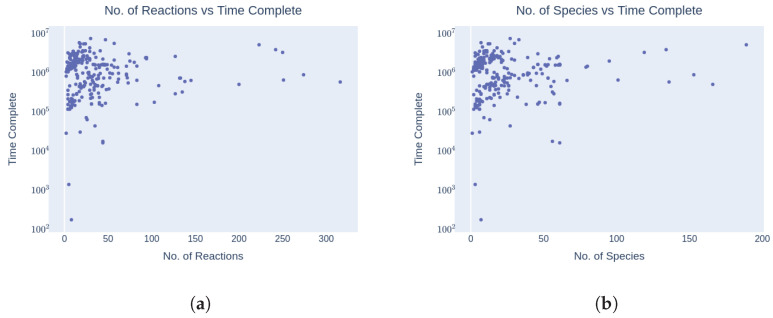

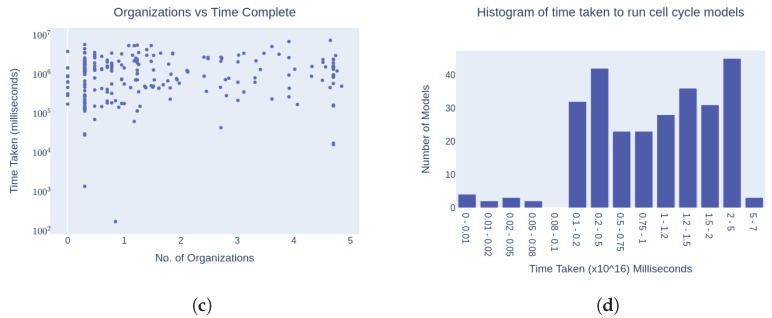
Time complexity: The range of computation time (between $10^{15}$ and $5\cdot 10^{16}$ milliseconds) required for the majority of models. The simulations were performed on a machine with an Intel Core i9-9300H CPU, a base clock speed of 2.4 GHz and 16 GB of DDR4 RAM. The operating system used was Windows 11 64-bit. (**a**) Number of reactions vs. time to compute organizations; (**b**) number of species vs. time to compute organizations; (**c**) number of organizations vs. time to compute organizations (milliseconds); (**d**) distribution of the number of models and time (milliseconds) required by them to compile.

Regarding time complexity, the majority of models required $10^{15}$ to $5\cdot 10^{16}$ milliseconds to run (Figure 10d).

Whereas the upper bound was due to certain self-defined termination conditions, the lower bound seemed to depend on the structure of the algorithm itself. In terms of time complexity, the majority of models required a runtime ranging from $10^{15}$ to $5\cdot 10^{16}$ milliseconds (Figure 10c). Interestingly, this range of computation times remained consistent across varying factors such as the number of species, reactions, and organizations (Figure 10a–c). This suggests that the algorithm’s sensitivity to the model’s structure is relatively low, indicating a brute force approach or lack of intelligence in the algorithm’s design (Figure 10a–c).

## 4. Conclusions

In this study, we examined a broad array of cell cycle models spanning various scales and theoretical frameworks. This comprehensive analysis provided insights into the key results regarding the persistence phenomenon within cell cycle research. The diverse organizational lattices explored underscored the complexity and variability inherent in biological systems.

This variability highlights a significant challenge: the concept of persistence in cell cycle research currently lacks a universal framework. There is no consistent, widely accepted approach among scientists, which impedes the development of standardized methodologies for studying cell cycle persistence. To address this challenge, we recommend further interdisciplinary collaboration and refinement of models to achieve a cohesive and universally recognized understanding of persistence in cell cycle dynamics.

The organizational model analysis presented here offers a valuable tool to address this ambiguity. Much like fixed-point analysis in dynamical systems, our framework provides a mathematically evaluated method to describe and compare various models. This approach can foster a deeper understanding of the principles governing cell cycle persistence and encourage more unified research efforts across the scientific community.

Our analysis revealed that models with complex compartmentalization, including those requiring more than two compartments, were prevalent across different model sizes. Therefore, we recommend a greater emphasis on compartmentalization in future cell cycle modeling approaches. Additionally, the persistence analysis showed a range of outcomes, from models where the entire cell cycle was modeled as a persistent system to those where only small subsystems (less than 10% of the total system) exhibited persistence.

The runtime complexity analysis indicated that the current algorithm for organizational analysis was relatively unsophisticated. Future research should explore advanced methods, such as those from artificial intelligence or machine learning, to improve computational efficiency and analytical depth.

Despite these limitations, the algorithm employed in this study was effective and user-friendly, providing valuable support for modeling cell cycle dynamics based on the framework we developed. We emphasize that continued advancements in methodology and interdisciplinary collaboration will be crucial in advancing our understanding of cell cycle persistence and improving modeling approaches in this field.

## Data Availability

The data were generated as explained here and can be obtained via Cell Cycle Analysis Github https://github.com/ArunJoseph19/Cell-Cycle-Analysis accessed on 13 September 2024.
